# Economic, Environmental and Health Implications of Enhanced Ventilation in Office Buildings

**DOI:** 10.3390/ijerph121114709

**Published:** 2015-11-18

**Authors:** Piers MacNaughton, James Pegues, Usha Satish, Suresh Santanam, John Spengler, Joseph Allen

**Affiliations:** 1 Department of Environmental Health, Harvard T.H. Chan School of Public Health, Landmark 409 West, 401 Park Drive Boston, MA 02115, USA; E-Mails: spengler@hsph.harvard.edu (J.S.); jgallen@hsph.harvard.edu (J.A.); 2 United Technologies Climate, Controls & Security, Syracuse, NY 13221, USA; E-Mail: James.F.Pegues@carrier.utc.com; 3 Psychiatry and Behavioral Sciences, SUNY-Upstate Medical School, Syracuse, NY 13210, USA; E-Mail: satishu@upstate.edu; 4 Industrial Assessment Center, Biomedical and Chemical Engineering Department, Syracuse University, Syracuse, NY 13210, USA; E-Mail: ssantana@syr.edu

**Keywords:** green buildings, energy and environmental costs, health, productivity

## Abstract

Introduction: Current building ventilation standards are based on acceptable minimums. Three decades of research demonstrates the human health benefits of increased ventilation above these minimums. Recent research also shows the benefits on human decision-making performance in office workers, which translates to increased productivity. However, adoption of enhanced ventilation strategies is lagging. We sought to evaluate two of the perceived potential barriers to more widespread adoption—Economic and environmental costs. Methods: We estimated the energy consumption and associated per building occupant costs for office buildings in seven U.S. cities, representing different climate zones for three ventilation scenarios (standard practice (20 cfm/person), 30% enhanced ventilation, and 40 cfm/person) and four different heating, ventilation and air conditioning (HVAC) system strategies (Variable Air Volume (VAV) with reheat and a Fan Coil Unit (FCU), both with and without an energy recovery ventilator). We also estimated emissions of greenhouse gases associated with this increased energy usage, and, for comparison, converted this to the equivalent number of vehicles using greenhouse gas equivalencies. Lastly, we paired results from our previous research on cognitive function and ventilation with labor statistics to estimate the economic benefit of increased productivity associated with increasing ventilation rates. Results: Doubling the ventilation rate from the American Society of Heating, Refrigeration and Air-Conditioning Engineers minimum cost less than $40 per person per year in all climate zones investigated. Using an energy recovery ventilation system significantly reduced energy costs, and in some scenarios led to a net savings. At the highest ventilation rate, adding an ERV essentially neutralized the environmental impact of enhanced ventilation (0.03 additional cars on the road per building across all cities). The same change in ventilation improved the performance of workers by 8%, equivalent to a $6500 increase in employee productivity each year. Reduced absenteeism and improved health are also seen with enhanced ventilation. Conclusions: The health benefits associated with enhanced ventilation rates far exceed the per-person energy costs relative to salary costs. Environmental impacts can be mitigated at regional, building, and individual-level scales through the transition to renewable energy sources, adoption of energy efficient systems and ventilation strategies, and promotion of other sustainable policies.

## 1. Introduction

Buildings account for 41% of US energy consumption, with nearly half of that energy usage coming from the commercial sector [1]. In office buildings, over half of the end-use energy expenditures are attributable to heating, ventilating, and cooling [2]. The environmental impact of these energy expenditures has been well documented; greenhouse gases emitted during power production are associated with climate change impacts including rising sea level, extreme temperatures, and more frequent weather events [3,4]. Emissions of sulfur dioxide (SO_2_) contribute to acid rain, which can damage sensitive ecosystems [5]. More important, however, are the downstream human health effects related to these environmental impacts. Elevated temperatures and droughts will increase the likelihood of heat-related illness and mortality [6]. Extreme weather effects also pose health and economic risks, especially in developing regions [7]. Emissions from power plants also have several direct health effects: (1) exposure to particulate matter, in particular SO_2_, increases the risk of respiratory and cardiovascular disease and (2) nitrogen oxides (NO_x_) cause airway inflammation and respiratory symptoms, especially in asthmatics [8,9].

At the building level, buildings managers are incentivized to reduce costs, which often is achieved by reducing ventilation rates. Similar incentives are not set for optimizing the health performance of buildings as occupant health is more difficult to characterize. Further, building managers tend to overestimate the energy costs related to ventilation. When asked the cost per occupant to double the ventilation rate from 20 cfm/person to 40 cfm/person and improve filtration from a minimum efficiency reporting value (MERV) 6 to a MERV 11 filter, building managers reported a perceived cost per occupant of $100 while the modeled estimates were consistently below $32 per occupant for all climate zones [10]. Consultants, tenants and owners also overestimated the costs of improved ventilation per occupant at $60, $115, and $80, respectively. Owners and building managers believe that tenants do not consider indoor air quality (IAQ) when leasing a space: 58% of respondents reported that 20% or less of their tenants take IAQ into consideration [10]. As a result, the cost of energy is often prioritized over IAQ and minimum required ventilation rates are met.

The guidelines that buildings operate under are by definition minimally acceptable. ASHRAE defined its original ventilation Standard 62 as “the minimum and recommended air quantities for the preservation of the occupants’ health, safety and well-being” [11]. In the initial standard, the minimum ventilation requirement was 10 cfm/person. Sick building syndrome (SBS) was first reported around the time of early standard adoption and coincided with improved sealing of building envelopes; occupants of poor performing buildings started reporting a wide range of symptoms including respiratory irritation, allergies, and headaches, which were later linked to the buildup of biological and chemical pollutants in the indoor environment [12].

In response, ASHRAE has since increased minimum acceptable ventilation rates under Standard 62.1 to approximately 20 cfm/person depending on the size and occupancy of the rooms within the building [13]. SBS symptoms and productivity losses have still been observed at this ventilation rate compared to higher ventilation rates. The prevalence of many SBS symptoms, such as throat/mouth dryness, feeling generally bad or good, and difficultly thinking, are reduced at ventilation rates above 20 cfm/person [14]. Recent research by our team also show cognitive improvements at 40 cfm/person compared to 20 cfm/person [15]. Absenteeism, productivity losses, and healthcare costs due to ventilation are estimated to have annual economic impacts in the hundreds of billions of dollars in the U.S. [16]. According to this analysis, a 5% change in productivity is equivalent to $125 billion in economic value based on the annual GNP of U.S. office workers, which is equivalent to $186 billion in 2015 dollars.

Sustainable or “green” design has sought improve occupant wellbeing in buildings while also reducing their environmental footprint. In 1990, Building Research Establishment Environmental Assessment Methodology (BREEAM) was founded as an international certification agency for green buildings. Three years later the Leadership in Energy and Environmental Design (LEED) rating system was established with a similar concept, focusing on U.S. buildings. Both agencies utilize design credits, which are subdivided into various sections, such as energy, water, and waste. Within each section there are required credits, which typically conform to local standards and guideline, and optional credits. To achieve a certain ranking, designers and architects can choose which optional credits to pursue. For example, LEED offers optional credits for both energy efficiency and increasing ventilation by 30%. In practice, the energy efficiency credits are preferentially chosen: only 40% of the new construction and 23% of the existing buildings rated under LEED v2009 obtained the enhanced ventilation credit. With advances in HVAC equipment such as energy recovery ventilators (ERVs), which significantly reduce energy use, it is possible to obtain credits for both energy efficiency and enhanced ventilation.

There is currently a lack of consensus about whether the energy costs and environmental impacts of increased ventilation outweigh the resulting health and productivity benefits. The burden of all four of these factors is estimated in a standard office building at 20 cfm/person (9.4 l/s/p), 27.6 cfm/person (13.0 l/s/p) (the ventilation rate to obtain the enhanced ventilation credit with LEED), and 40 cfm/person (18.8 l/s/p). We also test the effect of adding ERV to the higher ventilation scenarios. We then compare these scenarios to place the energy, environmental, health, and productivity factors into context.

## 2. Methods

### 2.1. Estimating Economic Costs of Enhanced Ventilation

Energy cost consequences are a function of local climate; local utility prices; building type, use and design; local building code requirements; ventilation rate; and HVAC system design. We used Carrier’s Hourly Analysis Program (HAP) to first calculate the annual energy consumption in kWh for fans, motors, pumps and chillers, plus natural gas in MCF (the volume of 1000 cubic feet (cf) of natural gas) for the hot water boilers for a range of scenarios, described in the following paragraphs. Energy use data was converted to kBtu/year (thousand Btu/year) so electric and gas consumption could be combined into a single value. Second, we estimated the annual per building occupant energy costs, in US dollars, associated with these energy costs based on local utility prices (Table 1) [17].

**Table 1:** Climate and fuel costs used for model inputs. Electricity generation fuel mix used for environmental impact assessment.

City Used in Study	Climate Zone	Summer Design		Winter Design		EIA State Average Price	
		Dry-Bulb	Coincident Wet-Bulb	Dry-Bulb		Electricity ($/kWh)	Gas ($/1000 ft^3^)
Austin, TX	2A–Hot, Humid	100 F (38 °C)	74 F (23 °C)	28 F (−2 °C)		0.0830	7.24
Charlotte, NC	3A–Warm, Humid	94 F (34 °C)	74 F (23 °C)	21 F (−6 °C)		0.0873	8.62
San Francisco, CA	3C–Warm, Marine	83 F (28 °C)	63 F (17 °C)	39 F (4 °C)		0.1457	7.05
Baltimore, MD	4A–Mixed, Humid	94 F (34 °C)	75 F (24 °C)	14 F (−10 °C)		0.1070	10.00
Albuquerque, NM	4B–Mixed, Dry	95 F (35 °C)	60 F (16 °C)	18 F (−8 °C)		0.0987	6.69
Boston, MA	5A–Cool, Humid	91 F (33 °C)	73 F (23 °C)	8 F (−13 °C)		0.1451	10.68
Boise, ID	5B–Cool, Dry	99 F (37 °C)	64 F (18 °C)	9 F (−13 °C)		0.0740	7.35
**City Used in Study**	**Energy Provider**	**Fuel Mix (%)**					
		**Renewables**	**Hydro**	**Nuclear**	**Oil**	**Gas**	**Coal**
Austin, TX	Austin Energy	7	0.2	12	0.8	45	34.8
Charlotte, NC	Duke Energy Carolinas	2	1.5	38.2	0.5	11.7	45.7
San Francisco, CA	City and County of SF	10.4	15.2	15.2	1.2	50.4	7.1
Baltimore, MD	Baltimore Gas and Electric	2	1	39.9	0.5	20.6	35.3
Albuquerque, NM	PNM Resource Inc.	3.4	6.2	17.5	0.1	33.4	39.5
Boston, MA	NSTAR	6.1	5.9	29.5	0.8	45.3	10.8
Boise, ID	Idaho Power Co.	6.8	43.6	3.4	0.3	14.3	31.3

For local climate, we modeled costs for seven U.S. cities that represent different climate zones. The climates ranged from hot/humid locales (Austin, TX, USA) to cool/dry locales (Boise, ID, USA), as seen in the dry-bulb and wet-bulb temperatures in these regions (Table 1). For building type, we were primarily interested in office buildings and selected the Department of Energy Medium Office Prototype as our template building [18]. This building type assumes a 53,661 sqft (4985 m^2^) floor area, three-story building with 268 occupants (200 sqft/person). We used ASHRAE 90.1-2010 Prescriptive Construction (code minimum) for construction type, which prescribes wall, roof and window assemblies, and light power density. For utility costs, we utilized the state average utility prices for each city in the model, using the Energy Information Administration electric and gas prices as of the date of this study.

For ventilation rate we modeled three rates, starting with the baseline condition as the ASHRAE Standard 62–2001 default minimum ventilation rate of 20 cfm/person of outdoor air. The 2001 edition of the standard was chosen for the baseline to be representative of existing building stock. Second, we calculated the outdoor airflow rate for the template building to obtain the LEED enhanced ventilation credit. The credit requires a ventilation rate 30% higher than ASHRAE Standard 62.1-2010, which is dependent on the occupancy and floor area of the building. For the template building this yields an approximate ventilation rate of 27.6 cfm/person. Last, we modeled the costs of enhancing ventilation to a doubling of the ASHRAE Standard 62-2001 minimum (40 cfm/person) based on findings of significant health and productivity benefits reported for this ventilation rate.

For the HVAC strategy, we used two air distribution systems that are typical for office buildings—variable air volume (VAV) and fan coil unit (FCU) systems. Both VAV and FCU are mature technologies with equal adoption by new and old buildings. For each system, we also evaluated the energy costs with and without an ERV to determine if an ERV mitigates the effects of increased ventilation rates.

A schematic of a typical VAV reheat system is provided in the Supplemental Information (Figure S1). This is a common HVAC application for offices that use a central station VAV air handling unit (AHU). Cooling is supplied to the AHU by an air-cooled chiller plant and heating is provided by a hot-water plant. Even in existing buildings, this HVAC system is capable of being modified to adjust the ventilation rate to 40 cfm/person, though an increase in chiller, boiler and/or AHU capacity may be required.

The second system we evaluated is 4-pipe fan coils (FCU) with a dedicated outdoor air system (DOAS) (Figure S2). This HVAC system is also a common application for office buildings. The DOAS conditions outdoor ventilation air and supplies it to the FCUs, which provide cooling and heating to rooms in the building. Similar to the VAV-reheat system, cooling is provided by an air-cooled chiller plant and heating is provided by a hot water plant. A limitation of the DOAS system is that it is designed for a certain ventilation rate (e.g., 20 cfm/person) and cannot readily accommodate significantly increased ventilation rates. The FCU system also has larger baseline energy costs compared to the VAV system for this building case study.

An ERV is a device that utilizes the energy in exhaust air from the building to heat or cool outdoor ventilation air entering the building. During heating seasons the exhaust air preheats and humidifies outside air, while the opposite happens during cooling seasons. This transfer of both sensible and latent heat saves energy that otherwise would be exhausted from the building, which reduces the energy that needs to be supplied by other elements of the HVAC system to maintain a specific set temperature and humidity.

For all scenarios, thermostat set points of 75 °F (23.9 °C) for cooling and 70 °F (21.1 °C) for heating with nighttime setbacks to 80 °F (26.7 °C) for cooling and 65 °F (18.3 °C) for heating. Humidity was not actively controlled in the model, but typically fell in the 40%–50% range for variable air volume (VAV) systems and 45%–60% range for fan coil unit (FCU) systems. The potential influence of this variability in humidity on cognitive function was not investigated.

### 2.2. Estimating Environmental Effects of Enhanced Ventilation

We used the energy usage estimates in the first part of the analysis described previously and the U.S. Environmental Protection Agency’s (EPA) Power Profiler to calculate the emissions for each scenario for the following air pollutants: nitrogen oxides (NO_x_), sulfur dioxide (SO_2_), and carbon dioxide (CO_2_). We used the most centralized zip code and most prevalent energy provider in each of the seven modeled cities. To provide another point of comparison, we also converted the energy usage information into emission equivalencies using EPA’s Greenhouse Gas Equivalencies Calculator. We estimated the emissions equivalent for the building energy consumption in terms of passenger vehicles per year. Passenger vehicles are defined as 2-axle 4-tire vehicles, including passenger cars, vans, pickup trucks, and sport/utility vehicles. In 2011, the weighted average combined fuel economy of cars and light trucks combined was 21.4 miles per gallon (9.1 km/L) [19]. The average vehicle miles traveled in 2011 was 11,318 miles (18,215 km) per year. In 2011, the ratio of CO_2_ emissions to total greenhouse gas emissions (including carbon dioxide, methane, and nitrous oxide, all expressed as carbon dioxide equivalents) for passenger vehicles was 0.988 [20]. The amount of carbon dioxide emitted per gallon of motor gasoline burned is 8.89 × 10^−3^ metric tons [21].

### 2.3. Estimating Health Benefits of Enhanced Ventilation

**Figure 1:**
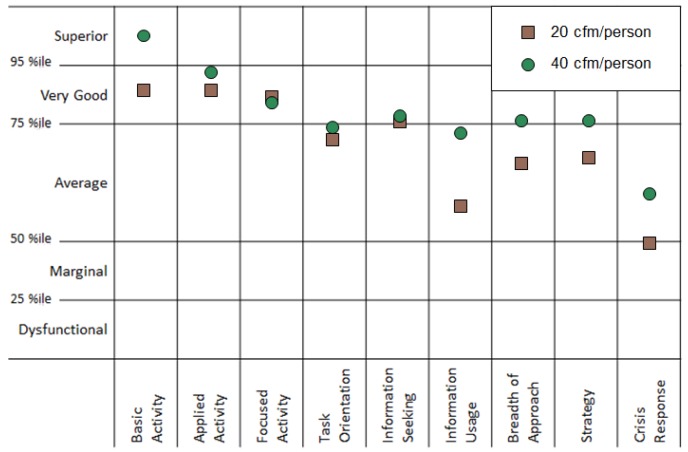
Average cognitive performance on the SMS tool of 24 participants in a green building at 20 and 40 cfm/person of outdoor air relative to normative data from ~70,000 people.

In a previous study of ventilation and cognitive function by our study team, 24 participants were exposed to 20 and 40 cfm of outdoor air per person on different study days [15]. At the end of each study day, they completed the Strategic Management Simulation (SMS) cognitive test, which measures decision-making performance across nine domains: basic, applied and focused activity level, task orientation, crisis response, information seeking and usage, breadth of approach and strategy. In this current paper, we used the participants’ scores from our previous study and plotted them against normative data of 70,000 previous SMS test takers to see the percentile shift from one ventilation condition to the next (Figure 1).

**Figure 2:**
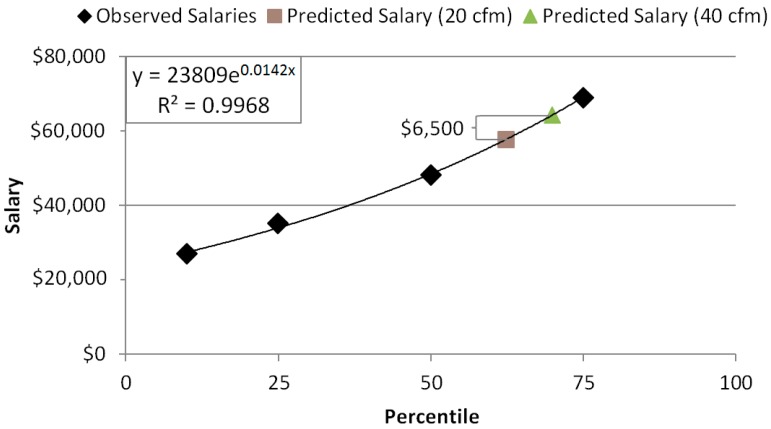
Observed salaries from the Bureau of Labor Statistics for common office occupations, regressed with an exponential function. This equation was used to interpolate salaries at the cognitive testing percentiles.

Data on salaries for various occupations was obtained from the Bureau of Labor Statistics (BLS). The 10th, 25th, 50th, 75th and 90th percentile salaries for management, business and financial operations, office and administrative support, computer and mathematical, architecture and engineering, legal, and sales occupations from May 2014 were used for analyses [22]. These occupation groups were selected to represent the U.S. office workforce and accounts for 57 million of the 135 million employments listed by BLS. Using averages weighted by the number of employments in each occupation group, mean salaries were computed for each percentile. The 90^th^ percentile was missing for management and legal occupations so it was excluded from subsequent analyses. These salaries were plotted and regressed using an exponential function (Figure 2).

## 3. Results

### 3.1. Energy Costs and Environmental Effects of Enhanced Ventilation

Energy costs are influenced by factors such as local climate, local fuel prices, building type, use, and design, ventilation rate and HVAC system. The modeled energy costs of enhanced ventilation are summarized in Table 2. Increasing ventilation rates 30% above the ASHRAE standard only costs $15 per occupant per year in Boston (cool, humid) and only $4–$7 per occupant per year in Albuquerque (mixed, dry) (results for other cities across the U.S. fall within this range). For a doubling of this minimum standard to 40 cfm/person, the costs for Boston and Albuquerque are $40 and $20 per occupant per year, respectively.

Adding an ERV to the system largely mitigates the energy costs of increasing ventilation. The ERV reduces the anticipated increase in energy costs by 60% when increasing ventilation to 40 cfm/person. At 30% above the ASHRAE standard, the ERV actually lead to cost reductions in three of the seven cities compared to the 20 cfm/person condition for VAV, and seven of seven for FCU (Table 2). San Francisco is a notable exception. San Francisco has moderate year-round temperatures. When an ERV is added, fan energy increases because fans must work harder to overcome the resistance to air flow through the ERV. In most locations this fan energy increase is offset by larger reductions in cooling and heating energy. For San Francisco, which also has high electricity costs compared to gas costs, the cooling and heating energy reductions are smaller than the fan energy increase, so the net effect of adding an ERV is a small energy cost increase. The FCU system has higher baseline energy costs and is typically more susceptible to ventilation changes, but it also is more affected by the addition of an ERV. As ventilation rates increase with the ERV in place, the FCU energy costs approach that of the VAV system (Table S1).

**Table 2:** Change in energy cost per occupant per year compared to conventional.

Ventilation Rate	Austin	Charlotte	San Francisco	Baltimore	Albuquerque	Boston	Boise
**Variable Air Volume**							
20 cfm/person	$0.00	$0.00	$0.00	$0.00	$0.00	$0.00	$0.00
27.6 cfm/person	$7.14	$7.29	$4.58	$10.42	$4.16	$12.03	$6.57
27.6 cfm/person + ERV	−$0.58	$0.42	$6.59	−$1.53	$3.77	−$0.82	$0.15
40 cfm/person	$23.07	$23.24	$15.73	$32.36	$14.34	$37.27	$20.78
40 cfm/person + ERV	$9.37	$10.55	$17.44	$11.21	$10.05	$14.06	$7.83
**Fan Coil Unit**							
20 cfm/person	$0.00	$0.00	$0.00	$0.00	$0.00	$0.00	$0.00
27.6 cfm/person	$7.31	$8.63	$8.69	$12.35	$7.77	$15.19	$9.19
27.6 cfm/person + ERV	−$0.18	−$3.46	−$0.05	−$7.29	−$0.72	−$8.35	−$6.77
40 cfm/person	$19.20	$22.70	$22.94	$32.42	$20.41	$39.87	$24.13
40 cfm/person + ERV	$8.32	$5.18	$10.22	$4.01	$7.88	$5.81	$1.00

The environmental impacts follow a similar trajectory as the energy costs. The environmental footprint of buildings can be reduced in five of the cities by increasing ventilation by 30% while simultaneously adding an ERV (Table 3). The cities with the largest percentage increase in CO_2_, SO_2_ and NO_x_ emissions are the ones with the lowest baseline energy usage. For example, San Francisco uses 44% less energy during the 20 cfm/person scenario than other cities (Table S2). The large percentage increase in energy usage at 40 cfm/person actually amounts to a small increase in emissions. At 40 cfm/person, these emissions correspond to between 6.2 and 18.9 additional cars on the road per year. Adding an ERV significantly reduces the number of additional cars (Table 4). At the highest ventilation rate, adding an ERV essentially neutralizes the environmental impact of enhanced ventilation (0.03 additional cars on the road per building across all cities). This is driven primarily by the benefits seen in buildings with FCU systems.

These results are dependent on the fuel mix in each city. Cities that rely primarily on combustion-based energy sources have larger environmental impacts. For example, 58% of the energy in Boise comes from renewables, hydro, or nuclear sources, compared to only 27.6% in Albuquerque (Table 1). The template building in Boise under 20 cfm/person of ventilation contributes to 3% less CO_2_ emissions than Albuquerque despite using 36% more energy on heating, cooling and ventilation each year.

**Table 3:** Percent increase in annual CO_2_, SO_2_ and NO_x_ emissions compared to conventional.

Ventilation Rate	Austin	Charlotte	San Francisco	Baltimore	Albuquerque	Boston	Boise
**Variable Air Volume**							
20 cfm/person	0%	0%	0%	0%	0%	0%	0%
27.6 cfm/person	14%	18%	25%	21%	17%	23%	21%
27.6 cfm/person + ERV	−1%	−2%	17%	−8%	0%	−10%	−7%
40 cfm/person	45%	55%	81%	63%	56%	67%	64%
40 cfm/person + ERV	17%	19%	61%	13%	18%	11%	13%
**Fan Coil Unit**							
20 cfm/person	0%	0%	0%	0%	0%	0%	0%
27.6 cfm/person	14%	16%	21%	18%	17%	19%	18%
27.6 cfm/person + ERV	−11%	−19%	−34%	−23%	−21%	−26%	−24%
40 cfm/person	36%	43%	56%	47%	44%	49%	46%
40 cfm/person + ERV	0%	−8%	−23%	−12%	−10%	−15%	−14%

**Table 4:** Number of additional cars per year on the road per building compared to conventional.

Ventilation Rate	Austin	Charlotte	San Francisco	Baltimore	Albuquerque	Boston	Boise
**Variable Air Volume**							
20 cfm/person	0.0	0.0	0.0	0.0	0.0	0.0	0.0
27.6 cfm/person	4.6	5.1	1.9	6.3	4.6	5.3	5.5
27.6 cfm/person + ERV	−0.5	−0.6	1.3	−2.5	0.0	−2.4	−1.7
40 cfm/person	14.5	15.7	6.2	18.8	14.6	15.6	16.7
40 cfm/person + ERV	5.6	5.6	4.7	3.9	4.7	2.6	3.5
**Fan Coil Unit**							
20 cfm/person	0.0	0.0	0.0	0.0	0.0	0.0	0.0
27.6 cfm/person	5.0	5.9	3.5	7.2	6.6	6.1	6.7
27.6 cfm/person + ERV	−4.0	−6.9	−5.3	−9.2	−8.2	−8.2	−9.1
40 cfm/person	13.2	15.5	9.0	18.9	17.3	15.8	17.6
40 cfm/person + ERV	0.1	−3.0	−3.6	−4.8	−4.0	−4.7	−5.2

### 3.2. Productivity Gains from Enhanced Ventilation

Figure 1 depicts the cognitive performance of the participants in our previous research on ventilation and decision-making performance [15]. These office workers performed above average in all domains. When ventilation was increased from 20 cfm/person to 40 cfm/person (the Green and Green + IEQ conditions respectively from that study), the participants increased from the 62nd percentile to the 70th percentile on average across all domains. Larger impacts were seen in basic activity, information usage, breadth of approach, strategy and crisis response than the other domains. When these percentiles were compared to the distribution of office worker salaries, they corresponded to a salary of $57,660 and $64,160 respectively, a difference of $6500 (Figure 2). When the occupational data was subsetted to management occupations, the difference in salaries at these percentiles was $15,500.

## 4. Discussion

Our motivation for this analysis stemmed from the observation that the public health benefits of enhanced ventilation have been researched and described for several decades, and our own recent research observed significant improvements in decision-making performance for office workers with enhanced ventilation, yet when we reviewed the prevalence of the selection of the enhanced ventilation credit in the leading green building rating system (LEED), we found that enhanced ventilation credit was pursued in only 40% of new buildings and 23% of existing buildings [15]. We hypothesized that one of the key barriers to more widespread adoption was the corresponding energy costs associated with increasing ventilation rates.

We found that the additional costs per occupant for enhancing ventilation rates were quite low; too low, in fact, to be a barrier for more widespread adoption. These costs are trivial (less than $40/year in the worst case scenario) when compared to the large improvements in cognitive function (greater than $6000/year) from increased ventilation. In our analysis, we examined the impact of including an ERV to offset energy usage and costs. As expected, energy usage costs dropped significantly with the use of these ERV systems in all U.S. cities. Most importantly, enhancing ventilation to 30% above the minimum, when paired with an ERV, led to cost savings in three of the seven cities in our model for VAV and seven of seven for FCU.

These findings are in agreement with Hamilton *et al.* that estimated annual costs from enhanced ventilation to be < $32 per person per year. In addition, they found cost perception may be a barrier to enhancing ventilation, despite the analysis that shows the actual costs to be low [10]. While the costs are low compared to productivity benefits, they do comprise a significant portion of building management budgets. The split incentive system, whereby building managers are responsible for energy costs while tenants are responsible for the cost of their employees, is a barrier to adoption as tenants cannot simply implement ventilation changes themselves. In addition, the health benefits of enhanced ventilation are not well-understood by most tenants as of yet.

The environmental costs represent another potential barrier to adoption of higher ventilation rates. While these costs are real, especially when magnified by all buildings in the U.S., the per building environmental impact on greenhouse gas emissions is not as impactful as the estimated benefits. These environmental impacts can be offset at three levels: individual, building, and system level. Pursuing other design features that promote alternative transportation options for individuals can reduce the environmental impacts from the building overall (incentivizing biking, preferred parking for electric cars, public transportation access). On a building-level, similar to the energy cost analysis, when ERVs are used the overall effect can be a net reduction in greenhouse gas emissions for the building. The environmental impacts can be reduced further through the use of more energy efficient HVAC systems, and, in new buildings, incorporation of advanced air distribution systems that deliver ventilation when and where it is needed to raise the effective ventilation per person, as opposed to the current approach of whole-building ventilation [23]. Last, on a systems-level, in cities with a greater percentage of use of non-combustion energy sources there is a lower environmental cost associated with enhancing ventilation.

The energy and environmental impacts are offset by the dramatic positive impacts that enhanced ventilation has on human health and productivity. In our recent study of office workers, when we mapped raw test scores onto normative data we found an eight percentile increase in decision-making performance when ventilation was increased from 20 cfm/person to 40 cfm/person, corresponding to a $6500 change in a typical office worker’s productivity. This is a conservative estimate of productivity gains and economic costs. First, the analysis in this paper only investigates cognitive impacts while in the office. The impacts of ventilation on other domains of health are well documented in the literature and lead to significant economic costs [24]. The risk of sick leave, illness, influenza and pneumonia are all elevated at lower ventilation rates and have additional productivity impacts (Table 5). With respect to sick leave, the cost per occupant is estimated to be an extra $400 each year at reduced ventilation rates [25]. The same study found this impact alone to dwarf energy costs by a factor of six among corporate workers. Second, as outdoor CO_2_ levels and temperatures rise as a result of climate change, the energy usage and IEQ of poor and high performing buildings will become increasingly disparate [26]. Third, our analysis only accounts for the direct costs associated with salaries; the employer cost for employee compensation is approximately 30% higher when considering benefits [22]. As higher paid positions have more expensive benefits, the reduction in costs to the company will be higher than estimated in this analysis. Fourth, the testing occurred in a LEED platinum building with low chemical concentrations. Even larger cognitive deficits were observed when chemicals were added to the space [15]. Enhanced ventilation in buildings with poor IAQ will lead to larger productivity gains than what was seen in this green environment. Lastly, the cognitive domains that have the highest correlations with other measures of productivity such as education level, salary at age, and number of employees supervised were the ones that had the largest improvements at higher ventilation rates [27]. The participants shifted from the 46.5th percentile to the 57th percentile on the information usage, strategy, and crisis response domains.

**Table 5:** Health impacts of ventilation rate in medium office prototype building (adapted from Fisk *et al.* [24]).

Reference	Outcome	Ventilation Rate (cfm/Person)		Relative Risk
		Low	High	
[25]	Short term sick leave	12.9	25.8	1.5
[28]	Illness all years	4.5	30	1.5
[28]	Illness 1983 data	4.5	30	1.9
[29]	Illness	48	120	2.2
[29]	Influenza	48	120	4.7
[30]	Influenza	15	45	3.1
[30]	Rhinovirus	15	45	2.1
[30]	TB	15	45	3.3
[31]	Pneumonia	20.4	30	2.0
[32]	SBS symptoms	8.5	42.4	5.0

These findings indicate that standard HVAC design and operation are not optimal for occupant health and decision making. New building construction should include ERVs and systems that can provide modifiable ventilation rates depending on outdoor air conditions. They should also invest in other ventilation strategies such as advanced air distribution systems and improved filtration, which reduces contaminants that may cause cognitive impacts. Green building architects and designers, which have the goal of improving occupant wellbeing while simultaneously reducing their environmental impact, should be particularly cognizant of ventilation strategies that can optimize these two factors. Credit-based rating systems should revisit their design requirements and properly incentivize these approaches.

Many existing buildings, on the other hand, have HVAC systems that are designed for a specific ventilation rate and may not be easily modified to increase ventilation rates. This limits the ability of building managers to make changes to the ventilation rate, even in light of the evidence presented in this paper. This limitation is similar for ERVs, which may not be easily installed into some existing systems.

Our analysis focused on one office type—the Department of Energy “Medium Office Type” template—and may not be applicable to other building types. In addition, this research and our previous research on cognitive function did not explicitly investigate thermal conditions, which may have independent productivity impacts. However, the modeling is straightforward and building owners for all buildings types could replicate our analysis for their specific building to estimate indoor conditions, energy costs, and environmental impacts. Last, energy costs fluctuate, but in our analysis model inputs are based on local costs and energy fuel mix as of the date that this manuscript was submitted. Any changes to the fuel mix or costs will change our estimated costs. Regardless, variation in the overall energy costs per occupant will be minor relative to their employment costs.

Several assumptions were made to derive the economic benefits of improved ventilation. First, we assume that the population of knowledge workers that took the cognitive testing was representative of the U.S. office workforce. 20% of that group held management positions compared to 12% in the BLS data, and 60% had professional occupations compared to 50% in the BLS data. Second, we assume a one percentile change in cognitive function corresponds to a one percentile change in value as an employee (e.g., someone who scores in the 62^nd^ percentile is salaried at the 62nd percentile). Previous work with the SMS tool has shown high correlations (>0.6) between cognitive scores and salary [27]. Third, this analysis demonstrates the competitive advantage to be gained by improving ventilation in comparison to workforce at large. As improvements to ventilation are adopted by a larger percentage of buildings, there will be an equilibration of wages to account for a generally more productive workforce.

## 5. Conclusions

The public health benefits of enhanced ventilation far exceed the per occupant economic costs in U.S. cities. Even with conservative estimates, the increased productivity of an employee is over 150 times greater than the resulting energy costs. Environmental costs are also relatively minor, but should be offset by the incorporation of energy recovery systems, advanced ventilation strategies, and other green building design strategies.

## Supplementary Files

Supplementary File 1

## References

[1] EIA (2015). How Much Energy is Consumed in Residential and Commercial Buildings in the United States.

[2] EIA (2008). Commercial Buildings Energy Consumpton Survey.

[3] Godlee F. (2014). Climate change. BMJ.

[4] Church J.A. (2001). How fast are sea levels rising?. Science.

[5] Abelson P.H. (1984). Effects of SO_2_ and NOx Emissions. Science.

[6] Anderson G.B., Bell M.L. (2011). Heat Waves in the United States: Mortality Risk during Heat Waves and Effect Modification by Heat Wave Characteristics in 43 USA Communities. Environ. Health Perspect..

[7] Cooney C.M. (2012). Managing the Risks of Extreme Weather: IPCC Special Report. Environ. Health Perspect..

[8] Spengler J.D., Sexton K. (1983). Indoor Air Pollution: A Public Health Perspective. Science.

[9] Levy J.I., Baxter L.K., Schwartz J. (2009). Uncertainty and Variability in Health-Related Damages from Coal-Fired Power Plants in the United States. Risk Anal..

[10] Hamilton M., Rackes A., Gurian P.L., Waring M.S. (2015). Perceptions in the U.S. building industry of the benefits and costs of improving indoor air quality. Indoor Air.

[11] (1973). Standard 62–73, Standards for Natural and Mechanical Ventilation.

[12] Redlich C.A., Sparer J., Cullen M.R. (1997). Sick-building syndrome. Lancet.

[13] Persily A. (2015). Challenges in developing ventilation and indoor air quality standards: The story of ASHRAE Standard 62. Build. Environ..

[14] Wargocki P., Wyon D.P., Sundell J., Clausen G., Franger P.O. (2000). The effects of outdoor air supply rate in an office on perceived air quality, Sick Building Syndrome (SBS) symptoms and productivity. Indoor Air.

[15] Allen J., MacNaughton P., Satish U., Santanam S., Vallarino J., Spengler J. Associations of Cognitive Function Scores with Carbon Dioxide, Ventilation, and Volatile Organic Compound Exposures in Office Workers: A Controlled Exposure Study of Green and Conventional Office Environments. http://ehp.niehs.nih.gov/wp-content/uploads/advpub/2015/10/ehp.1510037.acco.pdf.

[16] Fisk W.J., Rosenfeld A.H. (1997). Estimates of Improved Productivity and Health from Better Indoor Environments. Indoor Air.

[17] EIA (2015). State Electricity Profiles.

[18] DoE Commercial Prototype Buildings Models, B.E.C. Program. https://www.energycodes.gov/commercial-prototype-building-models.

[19] FHWA (2013). Highway Statistics 2011.

[20] EPA (2013). Inventory of U.S. Greenhouse Gas Emissions and Sinks: 1990–2011.

[21] EPA, DoT Light-Duty Vehicle Greenhouse Gas Emission Standards and Corporate Average Fuel Economy Standards, Final Rule. Federal Registry, 2010. Part II. http://www.gpo.gov/fdsys/pkg/FR-2010-05-07/pdf/2010-8159.pdf.

[22] BLS (2014). Occupational Employment Statistics.

[23] Melikov A.K. (2015). Advanced air distribution: Improving health and comfort while reducing energy use. Indoor Air.

[24] Fisk W.J., Olli S., David F., Yu J.H. Economizer system cost effectiveness: Accounting for the influence of ventilation rate on sick leave. Proceedings of the Healthy Buildings 2003 Conference.

[25] Milton D.K., Glencross P.M., Walters M.D. (2000). Risk of Sick Leave Associated with Outdoor Air Supply Rate, Humidification, and Occupant Complaints. Indoor Air.

[26] Holmes S., Reinhart C. (2013). Assessing future climate change and energy price scenarios: Institutional building investment. Build. Res. Inf..

[27] Streufert S., Pogash R., Piasecki M. (1988). Simulation-Based Assessment of Managerial Competence: Reliability and Validity. Personnel Psychol..

[28] Brundage J., Scott R.M., Lednar W., Smith D., Miller R. (1988). Building-Associated Risk of Febrile Acute Respiratory Diseases in Army Trainees. JAMA.

[29] Drinka P., Krause P., Schilling M., Miller B., Shult P., Gravenstein S. (1996). Report of an outbreak: Nursing home architecture and influenza—A attack rates. J. Am. Geriatr. Soc..

[30] Knibbs L., Morawska L., Bell S., Grzybowski P. (2011). Room ventilation and the risk of airborne infection transmission in 3 health care settings within a large teaching hospital. Am. J. Infect. Control.

[31] Hoge C.W., Reichler M.R., Dominiguez E.A., Bremer J.C., Mastro T.D., Hendricks K.A., Musher D.M., Elliott J.A., Facklam R.R., Breiman R.F. (1994). An epidemic pneumococcal disease in an overcrowded, inadequately ventilated jail. N. Engl. J. Med..

[32] Stenberg B., Eriksson N., Hoog J., Sundell J., Wall S. (1994). The sick building syndrome (SBS) in office workers. A case-referent study of personal, psychosocial and building-related risk indicators. Int. J. Epidemiol..

