# Supplementary material for: Economic, Environmental and Health Implications of Enhanced Ventilation in Office Buildings
Source: Int J Environ Res Public Health. 2015 Nov 18;12(11):14709–22. doi: 10.3390/ijerph121114709 (PMC4661675; doi:10.3390/ijerph121114709)
Supplement: Supplementary File 1 [file ijerph-12-14709-s001.pdf]

Economic, Environmental and Health Implications of Enhanced Ventilation in Office Buildings

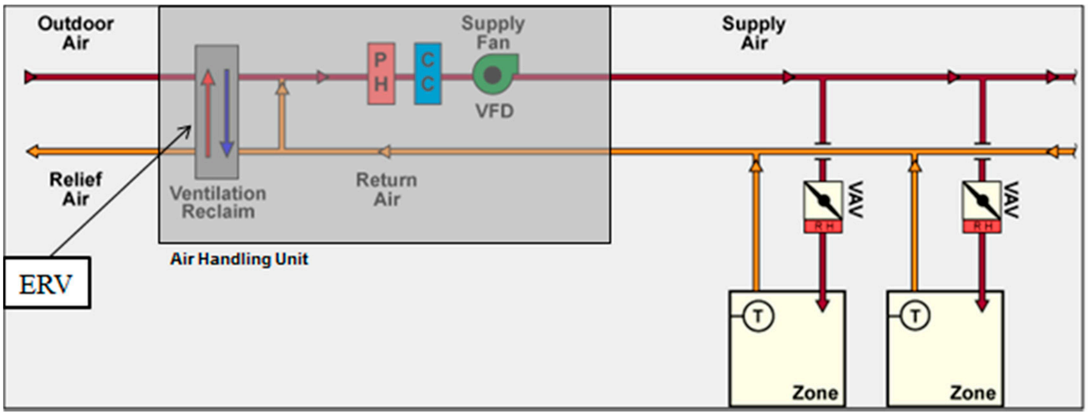

Figure S1. Variable air volume ventilation system schematic.

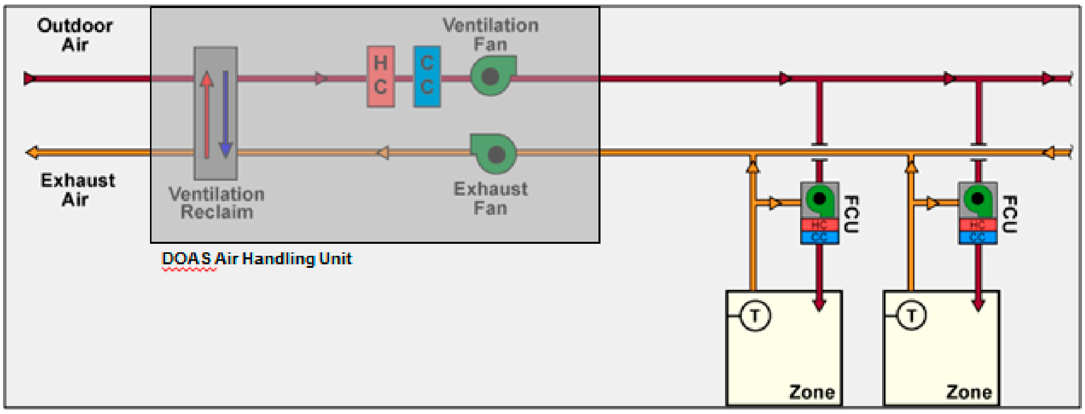

Figure S2. Fan coil unit ventilation system schematic.

Table S1. Simulated Annual HVAC Energy Cost ($/year). Electric energy cost for HVAC fans, motors, pumps, and chillers, plus gas cost for hot water boilers.

Ventilation Rate	Austin	Charlotte	San Francisco	Baltimore	Albuquerque	Boston	Boise
**Variable Air Volume**							
20 cfm/person	$16,142	$13,728	$7,143	$17,395	$12,521	$19,301	$11,054
27.6 cfm/person	$18,056	$15,683	$8,370	$20,187	$13,635	$22,526	$12,814
27.6 cfm/person + ERV	$15,987	$13,841	$8,908	$16,984	$13,532	$19,080	$11,093
40 cfm/person	$22,325	$19,957	$11,359	$26,067	$16,364	$29,290	$16,623
40 cfm/person + ERV	$18,652	$16,556	$11,816	$20,400	$15,215	$23,070	$13,152
**Fan Coil Unit**							
20 cfm/person	$17,077	$17,270	$16,639	$23,131	$18,600	$28,334	$17,134
27.6 cfm/person	$19,037	$19,583	$18,969	$26,440	$20,683	$32,404	$19,598
27.6 cfm/person + ERV	$17,029	$16,342	$16,625	$21,176	$18,408	$26,097	$15,319
40 cfm/person	$22,222	$23,354	$22,788	$31,820	$24,070	$39,020	$23,601
40 cfm/person + ERV	$19,308	$18,657	$19,377	$24,207	$20,712	$29,892	$17,403

Table S2. Simulated Annual HVAC Energy Use (Million Btu/year). Site electric energy use for HVAC fans, motors, pumps, and chillers, plus site natural gas use for hot water boilers.

Ventilation Rate	Austin	Charlotte	San Francisco	Baltimore	Albuquerque	Boston	Boise
**Variable Air Volume**							
20 cfm/person	892	894	422	1010	746	1088	1033
27.6 cfm/person	1018	1,053	527	1224	876	1339	1250
27.6 cfm/person + ERV	879	873	492	928	747	976	964
40 cfm/person	1291	1,388	763	1644	1,160	1820	1695
40 cfm/person + ERV	1045	1,068	680	1141	879	1209	1169
**Fan Coil Unit**							
20 cfm/person	1002	1145	879	1348	1130	1502	1498
27.6 cfm/person	1139	1330	1065	1591	1317	1784	1763
27.6 cfm/person + ERV	892	927	583	1037	897	1118	1135
40 cfm/person	1363	1633	1370	1984	1622	2242	2193
40 cfm/person + ERV	1006	1051	678	1187	1015	1283	1288

© 2015 by the authors; licensee MDPI, Basel, Switzerland. This article is an open access article distributed under the terms and conditions of the Creative Commons Attribution license (http://creativecommons.org/licenses/by/4.0/).
